# An intuitionistic approach for the predictability of anti-angiogenic inhibitors in cancer diagnosis

**DOI:** 10.1038/s41598-023-32850-8

**Published:** 2023-04-29

**Authors:** Syed Anas Ansar, Shruti Aggarwal, Swati Arya, Mohd. Anul Haq, Vikas Mittal, Fikreselam Gared

**Affiliations:** 1 Department of Computer Application, Babu Banarasi Das University, Lucknow, India; 2 Department of Computer Science and Engineering, Thapar Institute of Engineering and Technology, Patiala, Punjab India; 3 Department of Computer Science, College of Computer and Information Sciences, Majmaah University, Al Majmaáh, Saudi Arabia; 4 Department of Electronics and Communication Engineering, Chandigarh University, Mohali, India; 5 Faculty of Electrical and Computer Engineering, Bahir Dar Institute of Technology, Bahir Dar University, Bahir Dar, Ethiopia

**Keywords:** Cancer, Computational biology and bioinformatics, Health care

## Abstract

Malignant cancer angiogenesis has historically attracted enormous scientific attention. Although angiogenesis is requisite for a child’s development and conducive to tissue homeostasis, it is deleterious when cancer lurks. Today, anti-angiogenic biomolecular receptor tyrosine kinase inhibitors (RTKIs) to target angiogenesis have been prolific in treating various carcinomas. Angiogenesis is a pivotal component in malignant transformation, oncogenesis, and metastasis that can be activated by a multiplicity of factors (e.g., VEGF (Vascular endothelial growth factor), (FGF) Fibroblast growth factor, (PDGF) Platelet-derived growth factor and others). The advent of RTKIs, which primarily target members of the VEGFR (VEGF Receptor) family of angiogenic receptors has greatly ameliorated the outlook for some cancer forms, including hepatocellular carcinoma, malignant tumors, and gastrointestinal carcinoma. Cancer therapeutics have evolved steadily with active metabolites and strong multi-targeted RTK inhibitors such as E7080, CHIR-258, SU 5402, etc. This research intends to determine the efficacious anti-angiogenesis inhibitors and rank them by using the Preference Ranking Organization Method for Enrichment Evaluation (PROMETHEE- II) decision-making algorithm. The PROMETHEE-II approach assesses the influence of growth factors (GFs) in relation to the anti-angiogenesis inhibitors. Due to their capacity to cope with the frequently present vagueness while ranking alternatives, fuzzy models constitute the most suitable tools for producing results for analyzing qualitative information. This research’s quantitative methodology focuses on ranking the inhibitors according to their significance concerning criteria. The evaluation findings indicate the most efficacious and idle alternative for inhibiting angiogenesis in cancer.

## Introduction

Cancer is a disease that might affect anyone, irrespective of gender, age, or social status, and places a heavy strain on families, patients, and society. Cancer has been regarded as one of the cardinal causes of mortality worldwide. Despite the fact that angiogenesis is essential for a baby’s growth and beneficial for tissue repair, it is detrimental in the presence of cancer.As a necessity for both progression and metastasis of cancer, angiogenesis is not the only defining characteristic of the disease. Since non-angiogenic tumor growth has now been reported in tumors of several distinct organs in both human and preclinical animal models, this predicament is far from peculiar. Yet there is mounting evidence that some tumors can develop exclusively in a non-angiogenic form (even if they are hypoxic), while some tumors may comprise a hybrid of angiogenic and non-angiogenic regions. Angiogenesis can be triggered via various signaling pathways used by an assortment of proteins^1^. The process of angiogenesis involves the differentiation of endothelial cells (lining of the walls of the blood vessels), proliferation, and migration, for the development of new blood. A discrepancy between a beaucoup of anti- and pro- angiogenic endogenous parameters contributes to the worsening of the sickness. Angiogenesis is essential for the development of cancer since a blood supply is required for tumors to develop over a few millimeters in size. Apparently, tumors may trigger angiogenesis by releasing chemical signals that encourage its occurrence. Numerous angiogenic and anti-angiogenic factors control angiogenesis^2^. These anti-angiogenic medications have the ability to obstruct angiogenesis at a number of distinct stages. The transforming growth factor (such as VEGF, FGF, PDGF, etc.) exhibit positive regulatory action by interacting with the cell-extracellular matrix or activating cell surface receptors, but in a few cases, any deviations from normal function lead to a variety of developmental abnormalities^3^. Even, sometimes the angiogenesis signals might be unbalanced, leading to excessive blood vessel formation that can result in abnormal circumstances or disease that help in tumor growth. The main regulatory signaling proteins that control the growth of cancer cells and their metastasis are referred to as RTKs (Receptor tyrosine kinases). Research has demonstrated that RTKs play a significant role in the initiation and development of many different forms of cancer, in addition to being important regulators of normal cellular activities^4^. Cellular signaling pathways, mediated by RTKs, regulate many physiological functions, including transcription factors, metabolic control, angiogenesis, proliferation, cell growth, and survival. When a receptor, as with a GF, binds to an RTK, the signaling pathways are initiated. Intricate pessimistic feedback chain inhibits activation of the RTK signaling pathway by diminishing signaling from activated receptors^5^.

**Figure 1:**
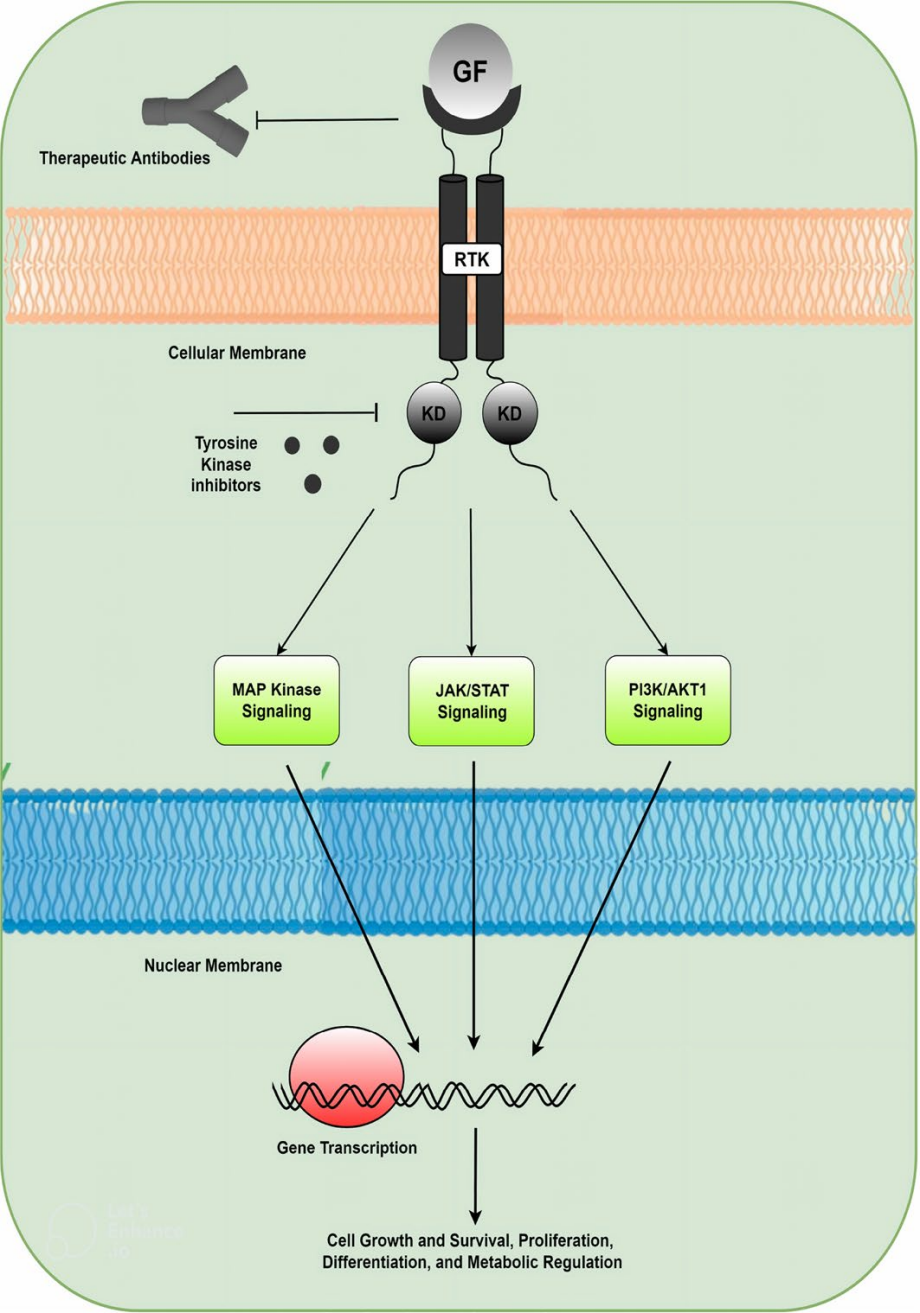
Binding of Growth Factor (GF) to RTKs.

When a growth factor (like VEGF or PDGF) binds to an RTK, the receptor tyrosine signaling pathway is activated, which usually results in the dimerization of the 2 receptor monomers. By being phosphorylated within their kinase lobes, the receptors are stimulated. Several other cascade pathways are activated after the receptor is switched on, particularly Flt1/ KDR or Flk1 signaling, FGFR signaling, and PDGFR $\alpha$/$\beta$ kinase signaling. The therapeutic responsive nodes in the pathway are indicated specifically in Fig. 1. Several RTK targeting medicines have been employed as first- or second-line therapies in oncology for various cancers throughout the past 20 years. This study examined several RTKs, namely, VEGFR (VEGF receptor), FGFR (FGF receptor), and PDGFR (PDGF receptor) along with its inhibitors. Inhibitors have become a vehement platform technology for developing decisive medicines for the treatment of cancer. Although it plays a significant role in precision oncology, numerous inhibitors including Bay73-4506, ABT-869, SU 5402, and others have also been examined as potential pharmaceuticals and a slew of medications have been licensed as a consequence of their remarkable explicitness and low toxicity. The anti-angiogenic inhibitors have been identified as a potential cure for the future, but there are various instances that lead to failure. Seeing as in the majority of solid tumors, VEGF and its receptors are upregulated, which stimulates endothelial cell migration and proliferation, resulting in the development of new blood vessels from existing ones^6^. Howbeit, studies revealed that PDGF-B mutant mice models developed vascular anomalies without pericytes recruitment, demonstrating their significance as regulators of mural cell recruitment which is evident from the suggestions that in most cases anti-angiogenic and anti-tumor inhibitors enact a significant role in the treatment of cancer^7,8^. Utilizing angiogenesis inhibitors (anti-angiogenic drugs), angiogenesis can be addressed as part of the therapy. Alongside, combination medicines based on inhibitors are also being explored for treating cancer. The cancer cells constantly develop transformational mechanisms through which another alternative pathway is elevated in case any of the transformational channels is inhibited.

Angiogenesis inhibitors are distinct from other cancer treatment substances as they prevent the development of blood vessels responsible for the growth of tumors rather than tumor cell proliferation^9,10^. These inhibitors disrupt different phases of the formation of blood vessels in a myriad of ways. Most of them are VEGF-specific monoclonal antibodies that identify and bind with them. These medications prevent VEGF from activating the VEGF receptor when it is bound to them. Certain angiogenesis inhibitors bind to VEGF, FGF, or PDGF and their receptors along with additional receptors found on the endothelial cells (EC) surface or may be to some extra proteins in the subsequent signaling trails, preventing their functionalities. Various immunomodulatory medicines or substances that boost or decrease the immune system likewise have anti-angiogenic characteristics and are angiogenesis inhibitors. Fuzzy models are the ideal tools for generating outcomes when assessing qualitative data since this model have the ability to deal with the frequently present ambiguity while ranking alternatives. This study examines the various alternatives to identifying the optimal anti-angiogenic inhibitors using an intuitionistic approach i.e., PROMETHEE- II multi-criteria analysis technique.

### Research contributions

This study provides insight into angiogenesis and its factor that leads to unbalanced signals, leading to excessive blood vessel growth. Angiogenesis, which is pertinent for the development of tumor growth, maintenance, and metastasis, is often started from the capillaries. Several forms of cancer can be treated with anti-angiogenic medicines that use either antibodies or tyrosine kinase inhibitors. The main vision of this research is a comprehensive study of the growth factors and their receptors, analyzing the relationship between anti-angiogenic inhibitors with VEGF, FGF, and PDGF receptors; determining the ranking of selected inhibitors by employing the PROMETHEE II technique. Using the PROMETHEE II methodology, a number of cancer anti-angiogenic inhibitors and their receptors were investigated to predict the finest decision results.

### Paper organization

The manuscript has been designed in the most simplistic and understandable way, which aims to convey a broad knowledge of the topic. The paper has been bifurcated into 6 sections, listed below: “Analysis of contemporary literature” section reviews the contemporary literature in this field. “Anigiogenic growth factors and inhibitors” section discusses the growth factors of cancerous angiogenesis and potential inhibitors to inhibit their receptors. The experimental methodology and results of PROMETHEE-II are detailed in “Methodology” section. Later, “Numerical analysis” section analyzes the quantified data utilizing above mentioned methodology and concludes the paper in “Conclusion” section.

## Analysis of contemporary literature

For therapeutic approaches to cancer, there has been a variegated of prior studies applying qualitative methodologies and a multiplicity of strategies. To analyze various factors of cancer, several researchers have utilized MCDM (Multiple criteria decision-making) technique to evaluate results based on the criteria. In this sphere, the researchers have scrutinized the following scientific papers:

Sekar et al. (2022), have investigated the patients’ risk factors for LC (Lung Cancer) and suggested that they should receive treatment as a high priority for their longevity. As per the proposed ranking methods, the tentative fuzzy- VIKOR method is employed to evaluate the extremity of Cancer patients’ disease. The authors divulge that everyone who inhales the smoke of another person’s cigarette (second-hand smokers) will likewise suffer consequences. This study exhibits that VIKOR has a remarkable result in locating both positive and negative traits when employed and studied in decision-making^11^.

Mustapha et al. (2022), proposed a novel approach for screening breast cancer using MCDM (Multiple criteria decision-making) and supervised learning. In order to evaluate machine learning (ML) models more thoroughly, this study will use a cutting-edge methodology that blends artificial intelligence (AI) and an MCDM process for more robust results with the PROMETHEE. This research reveals that the Support Vector Machine is ranked to be the most advantageous approach for the timely identification of breast cancer, having obtained a net outranking flow of 0.1022. The findings of the study indicate that this method can be used to choose the optimal machine learning model while still achieving the desired goals. As a result, the decision-maker has the choice to add new criteria to the decision-making process^12^.

Ozsahin et al. (2021) explored several advanced brain cancer treatments, including surgery, radiotherapy, targeted drug treatment, chemotherapy, and tumor-targeting. This research aims to determine the optimal course of treatment based upon different predetermined principles. MCDM technique, i.e., PROMETHEE is used to analyze the various factors of brain cancer therapy based on variables that may have an impact on how well they work. Tumor-treating fields are a better strategy than other cutting-edge brain cancer therapy methods, according to evaluation results based on the criteria undertaken by the researcher^13^.

Feola et al. examined the current and potential clinical uses of combining OV (Oncolytic Viruses) with other cancer immunotherapies. According to them, cancer immunotherapy depends on using the immune system of patient to target particular anti-tumor reactions and eventually destroy cancer. OVs are a unique type of cancer immunotherapy that has surfaced among various therapeutic modalities. OVs can function as in situ therapeutic cancer vaccines because the immunogenic cancer cell death driven by OVs triggers the secretion of tumor antigens, which in succession stimulates anti-tumor antibodies. They may be employed in conjunction with other cancer immunotherapeutic strategies, including CAR-T cells and immune checkpoint inhibitors^14^.

According to Drozdz et al., Traditional cancer therapeutic approaches, such as radiotherapy, chemotherapy, and immunotherapy, have significant limitations. The capability of obligatory or facultative anaerobic microbes to infiltrate and proliferate in hypoxic parts of tumors has led the way to the generation of bacteria-mediated cancer diagnostics as a powerful technique for the cure of cancer. Through a number of processes, such as the generation of toxins, anaerobic lifestyle, and interaction with anti-cancer medications, anaerobic bacteria promote the retrogression of tumors and suppression of metastases. These qualities might be applied as an addition to current cancer treatments^15^.

According to Sosnowska et al, a number of pathological disorders, including aggressive cancer, are brought on by the extracellular matrix (ECM) degrading because it alters the physicochemical characteristics and dysregulates the connections between cells. Being a material that is easily functionalized and biocompatible, carbon nanofilm can be utilized to impersonate ECM formation, altering the deportment of cancer cells to make them behave as healthy cells. Researchers carried out experiments in vitro with HS-5 cells (used as a control) as well as C3A and HepG2 cancer cells. The fullerene C60 nanofilm was created using an aqueous solution. Confocal, light, atomic and electron force microscopy were used to assess the morphological characteristics of cells grown on C60 nanofilms. It was discovered that C60 nanofilm was biocompatible, non-toxic, and replicated the niche/ECM of cells. However, the electromechanical frequency response from C60 nanofilms generated a habitat that impacted the cycle of cell and decreased cell growth, indicating that C60 nanofilms would be a viable alternative element for the cancer cell’s niche^16^.

Djatna et al. postulate that one of the impediments to recognizing stroke disease is the inability to use a powerful analysis tool to find crucial stroke data from a large volume of data that incorporates hidden linkages and patterns. For the purpose of diagnosing the various forms of stroke disease, they suggested an intuitionistic, fuzzy-based decision tree. Implementing the method entails converting collected data into an intuitionistic fuzzy set. The Hamming Distance, a primary prerequisite for intuitionistic fuzzy entropy, is used to compute the outcome of intuitionistic fuzzy. Based on the knowledge gained from variables, the results pick the root with the node in the construction of the decision tree structure. The outcomes of the simulations demonstrate that the method effectively identifies 20 variables that directly impact stroke^17^.

Ozsahin et al. (2017) evaluated numerous cancer treatments, namely radiotherapy, chemotherapy, hormone therapy, surgery, and immunotherapy using the Fuzzy PROMETHEE technique. This multi-criteria analysis technique evaluated the good decision results of different cancer treatments. According to this study, the proposed methodology presents the finest decision-making issues of substantial alternatives. The outcomes of this ranking enable decision-makers to choose or enhance the treatment protocols in order to apply the MCDM method to several kinds of cancer treatment strategies. Comparing this method to other decision-making processes, it has been found to be much more effective in several domains^18^.

Anti-angiogenic inhibitors have been the subject of numerous investigations, while a smaller number of studies have been performed to determine and rank the most effective anti-angiogenesis inhibitors. As Ozsahin et al. utilized the Visual PROMETHEE decision lab program and applied a Gaussian preference function for each criterion. The six therapeutic choices explored in their study were ranked from most to least effective using the PROMETHEE approach which partially ranks alternatives, whereas PROMETHEE-II has proven to provide a complete ranking of the alternatives.

## Anigiogenic growth factors and inhibitors

### Angiogenic growth factors

**Figure 2:**
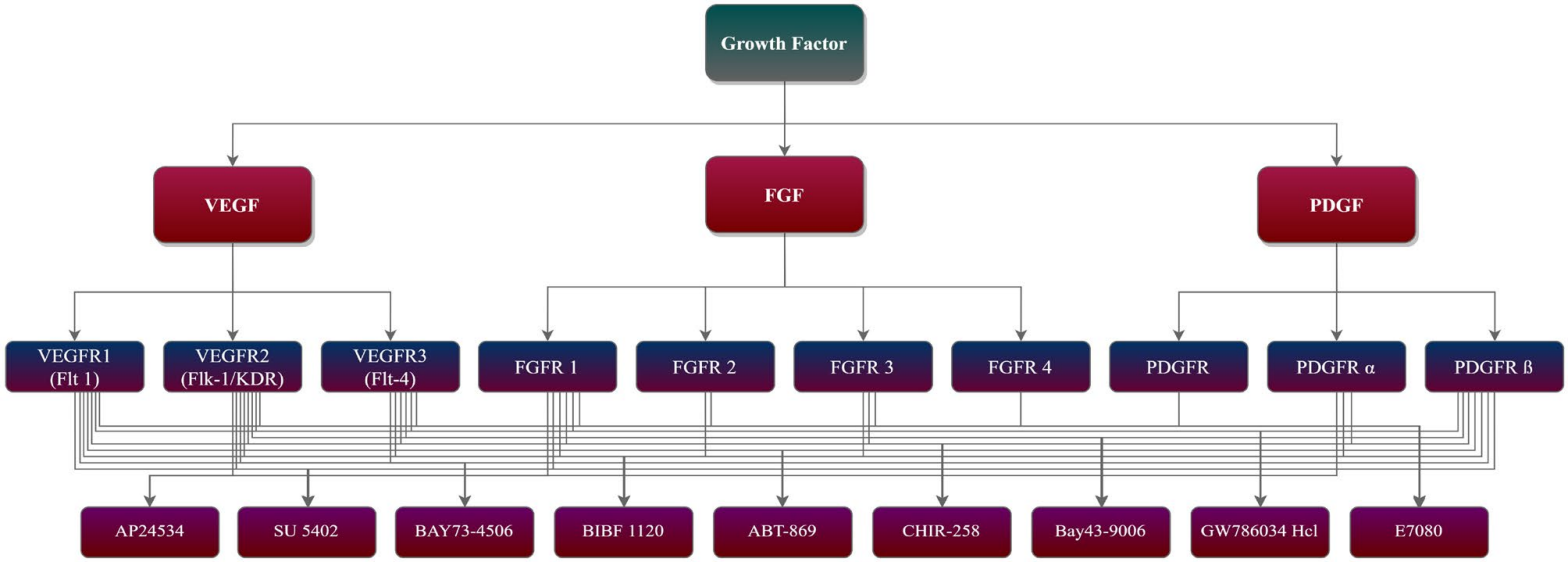
Hierarchical Representation of Angiogenesis GF along with Receptors and Inhibitors.

An important function in the development of blood vessels is played by a group of molecules known as angiogenic growth factors. Several distinct cell types are engaged during the angiogenic activity, featuring ECs and circulating endothelial progenitor cells, vascular smooth muscle cells, stromal cells, and pericytes. These interactions take place via secreted factors, including VEGF, PDGF, FGF, and angiopoietins, as well as cell-extracellular matrix (ECM) interactions. This study delineated three key factors shown in “Fig. 2”, expounded on them by examining their receptors, and identified generic inhibitors that bind to and inhibit those receptors in Table 1.

#### VEGF

Sengar and Dvorak (1983) were the first to functionally describe VEGF-A, and thenceforth, the VEGF class of growth factors has gained widespread recognition as an essential angiogenic factor. VEGFR1, VEGFR2, and VEGFR3 are a family of RTKs that modulates the signaling caused by these GFs. VEGF is a signal protein that stimulates the expansion of multiple blood vessels, which are released by a wide range of cells. As VEGF is a subfamily of GFs, each of these kinases has various degrees of selectivity for different VEGFs. Cytokines, numerous polypeptide growth factors, and hormones bind to them with great fidelity on the cell surface. Around 20 distinct classes of RTK have been identified, so far^19^.

#### FGF

The FGF family constitutes the largest family of growth factors, with 23 members with striking sequence similarities^20^. Fibroblast growth factor receptors (FGFRs) are all bound by FGF1 through FGF1 members. Both FGF1 and FGF2 are sometimes referred to as acidic and basic fibroblast growth factors, respectively. FGF1 and FGF2 signaling in angiogenesis is predominantly mediated by their extracellular linkages with FGFR1 and FGFR2^21^. FGFs have been demonstrated to activate FGFRs, which in turn affects ECs survival, migration, and proliferation. They are believed to be one of the primary elements implicated in the early stages of angiogenesis. The family of RTKs known as FGFRs is distributed on the cell membrane and is indispensable for both the development and maturation of cells. Numerous malignancies, including hepatocellular carcinoma, ovarian cancer, lung adenocarcinoma, and urothelial carcinoma, have been linked to FGFR dysregulation. They have been recognized as a prospective therapeutic target because of their functional significance for the treatment of several malignancies.

#### PDGF

Strong mitogenic factors (mitogens), such as PDGFs, control cell division and growth in fostering neural systems and connective tissue. PDGFs are released by a wide array of cell types, involving inflammatory cells, endothelial, glial, epithelial, and platelets. In general terms, pericytes, fibroblasts, glial cells, mesangial cells, and smooth muscle cells are among the cells derived from mesoderm targeted by PDGFs. PDGF$\alpha$, PDGFR$\beta$ are two different class III RTKs that have two corresponding transmembrane receptors^22^.

**Table 1:** Receptors of Angiogenic Growth Factors with their Synopsis.

Growth Factor	Receptors	Synopsis
VEGF (GF1)	VEGFR-1(Flt1)	Angiogenesis, cell migration, gene expression, and vascular permeability are all stimulated by VEGF-A’s binding with and activation of VEGFR-1 and VEGFR-2^23^.
		The formation of hematopoietic stem cells depends on VEGFR-1 or FLT-1.
		VEGFR-1 also serves as a fake or false receptor, inhibiting the linkage of VEGF to VEGFR-2.
		VEGFR-1 is an impaired RTK.
	VEGFR-2(KDR or Flk1)	The majority of known cellular reactions to VEGF are regulated by VEGFR-2^24^.
		VEGFR-2 is a kinase with high reactivity.
		Vascular ECs and hematopoietic stem cells produce the 210-230 kDa glycoprotein known as VEGFR-2, which binds VEGF-A^25,26^.
		Various downstream pathways that are over-activated in certain cancers require active auto phosphorylated VEGFR-2^25^.
	VEGFR-3	In the presence of VEGF-D and VEGF-C, VEGFRR-3 mediates lymph angiogenesis.
		In the corpus luteum of several species, including cattle, buffalo, and monkeys, contains VEGFR-3 in lymphatic ECs^27^.
		Endothelial cell migration, survival, and proliferation are all influenced by VEGFR3, which also controls angiogenic sprouting^22^.
FGF (GF2)	FGFR-1	Breast, lung, hematological, and other forms of cancer are hypothesized to be influenced by epigenetic alterations and somatic mutations for the expression of the FGFR1 gene.
		Non-small-cell lung cancer patients who had FGFR1 gene amplification (>=4 copies) ranged in frequency from 9 to 22$\%$.
		Ponatinib and Dovitinib efficiently address the FGFR1 mutation caused by over-amplifications of genes in lung and breast cancer^28^.
	FGFR-2	It particularly in blood arteries and bone plays crucial functions in tissue healing and the development of the embryo.
		These receptors similar to other FGFRs, send signals by dimerizing with their ligand, which triggers the RTK domains to start a cascade of intracellular signals.
		These signals drive cell division, proliferation, and differentiation at the molecular level^29^.
	FGFR-3	As a prevalent therapeutic target in bladder urothelial carcinoma (BLCA) with FGFR3 mutations, FGFR3 is often altered across a variety of cancer types^30^.
		By stabilizing ossification, the FGFR3 protein contributes to bone development^31^.
		Bladder cancer is often linked with FGFR3, FGFR3-TACC3, and FGFR3-BAIAP2L1 fusion protein mutations; however, certain FGFR3 mutations are also associated with improved prognosis. Therefore, in order to treat bladder cancer, FGFR3 may prove to be a potential therapeutic target^32^.
	FGFR-4	The protein encoding performed through this gene controls multiple processes such as glucose absorption, lipid metabolism, phosphatehomeostasis, synthesis of bile acid, vitamin D metabolism, cell differentiation, migration, and proliferation.
		Three immunoglobulin-like domains, a cytoplasmic tyrosine kinase domain, and one hydrophobic membrane-spanning segment make up the extracellular part of this protein, which is also present inside cells.
		A series of downstream signals are triggered by the protein’s extracellular region’s interactions with fibroblast growth factors, which eventually affect mitogenesis and differentiation^33^.

Growth Factor	Receptors	Synopsis
**PDGF**	PDGFR	This receptor belongs to RTKs for PDGF families, a subclass of cell surface receptors.
		In PDGFR, A and B are subunits of PDGF, that have a significant role in regulating cancer cells, cellular differentiation, development, and cell growth^34^.
	PDGFR-$\alpha$	It can only be triggered by PDGF-B and -D.
		There have been reports of PDGFR- $\alpha$, being dysregulated in a number of cancers, like breast cancer, glioblastoma, hepatocellular carcinomas, ovarian cancer, and pancreatic cancer^35^.
	PDGFR-$\beta$	PDGFR- $\beta$ binds PDGF-A, -B, and -C.
		Stromal cells primarily express PDGFR- $\beta$ in epithelial malignancies.
		Although high PDGFR- $\beta$ expression has been correlated to poor prognosis in a broad range of malignancies, its prominence in colorectal cancer (CRC) is still unclear^22^.

### Angiogenic inhibitors

Inhibitors of angiogenic have substantial therapeutic potential to prevent angiogenesis and are doable to amalgamate angiogenic inhibitors with other anticancer medications or use them alone as monotherapy. Dr. Folkman originated and disseminated the concept of anti-angiogenic therapy with the creation of endostatin^36^. Diverse approaches have led to the creation of antitumor agents. Thus far, several inhibitors have been addressed in Table 2, alongside their chemical structures and aliases.

**Table 2:** Elucidation of Angiogenic Inhibitors along with Chemical Structures.

Inhibitors	Alias	Chemical Structure	Elucidation
AP24534 ($In_1$)	Ponatinib		It can inhibit cell growth and angiogenesis as well as cause cell apoptosis by inhibiting RTK (PDGFR, VEGFR2, and FGFR1) activities^37,38^.
SU 5402 ($In_2$)	su5402		SU5402 is an oxindole in which the 3-(2-carboxyethyl)-4-methyl-1H-pyrrol-2-yl group is replaced for one of the methylene group’s hydrogens.
			It has been shown to possess an inhibitory concentrationIC50 for 20 nM-VEGFR2, 30 nM-FGFR1, and 510 nM-PDGFR$\beta$^39^.
BAY73-4506 ($In_3$)	Regorafenib		It is an orally ingested secondary metabolite with antiangiogenic and anticancer properties and inhibits PDGFR$\beta$, RET, VEGFR1/2/3, RET, Raf-1, and Kit^40^.
BIBF 1120 ($In_4$)	Nintedanib		Upon ingestion, it preferentially interacts with and inhibits VEGFR1/2/3, FGFR1/2/3, and PDGFR $\alpha$/$\beta$, potentially leading to endothelial cell death, diminished tumor vasculature, restriction of tumor cellular transformation, and anti-fibrotic action in pulmonary fibrosis^41^.
ABT-869 $(In_5)$	Linifanib		It is an effective, targeted antagonist of the VEGFR and PDGFR families that acts to slow the growth of tumor by inhibiting the establishment of neovascularization that deliver nutrition and oxygen to the tumor and inhibiting vital angiogenic signal transduction^42–44^.
CHIR-258 $(In_6)$	Dovitinib		It is an RTK antagonist with putative anti-cancer effects. It binds to and prevents the activation of type III-V RTKs, particularly FGFR3, VEGFR1-3, FLT3, c-Kit, CSF-1R, and PDGFR$\alpha$/$\beta$, which support tumor cell cycle progression in some cancerous cells^45,46^.
Bay43-9006 $(In_7)$	Sorafenib		Late-stage renal and hepatocellular carcinoma that cannot be removed surgically, and a typical thyroid carcinoma that has returned or has expanded to other areas of the body, are all treated with sorafenib.
			It prevents angiogenesis in tumor growth by inhibiting c-Kit, VEGFR/2/3, FLT3, and PDGFR$\beta$, signal transduction^47^.
GW786034 HCL $(In_8)$	Pazopanib Hydrochloride		It is an HCl solution of a biomolecular antagonist that possesses the ability to exert anticancer effects on several protein tyrosine kinases.
			It specifically inhibits VEGFR1/2/3, FGFR1, c-Kit, c-Fms, and PDGFR, which may prevent tumors with high concentrations of these receptors from undergoing angiogenesis^48^.
E7080 $(In_9)$	Lenvatinib		It is an anti-cancer inhibitor that can potentially treat several forms of thyroid cancer and other malignancies.
			The receptors VEGFR1/2/3, KIT, FGFR1/2/3/4, PDGFR, and RET are all targets of this Eisai Co-developed multiple kinase inhibitor^49,50^.

## Methodology

The PROMETHEE is an arithmetical ranking methodology, invented in 1985 by Brans and Vincke. This methodology has undergone many versions, involving PROMETHEE I, II, III, IV, V, VI, Cluster, TRI, and Fuzzy etc. Depending on the issue, each technique is applied differently. The interactive nature of PROMETHEE II and its potential to identify and rank alternatives that are intricate and challenging to contrast are the reasons, the authors have selected this technique^51^. A pair-wise assessment of each criterion’s alternatives serves as the foundation for the PROMETHEE II principle that possesses additional qualities including stability, simplicity, and clarity. This indicates that a variety of criteria must be optimized or limited in order to assess the alternatives^52^. No matter how the alternatives respond to other criteria, each criterion should be able to discriminate between them^53^. In essence, PROMETHEE II endows an extensive ranking of alternatives though, in the MCDM approach, the Decision Matrix (DM) should identify alternatives, weights, and evaluate scoring criteria to quantify the relationships among various alternatives using a fuzzy verbal scale that indicates how highly they rank in relation to one another^54^. The growth factors of angiogenesis or criteria are **GF1**, **GF2**, and **GF3**, including $In_1$, $In_2$, $In_3$, $In_4$, $In_5$, $In_6$, $In_7$, $In_8$, and $In_9$ are alternatives. The PROMETHEE-II will be thoroughly necessary to be incomparable and examine and comprehend MCDM^55^. The criterion of the optimum solution by the utility function minimizes the MCDM issue.

The complete restructuring of MCDM’s structure amplifies the utility function. Ranking relations are the foundation of the PROMETHEE-II technique. It does not offer an identical alternative for every criterion. The PROMETHEE-II method’s inclination architecture is primarily centred on pair-wise individualized comparing among each alternative. The distinctions between the two criterion alternatives are taken into account in the PROMETHEE-II technique. The researcher picks the preferences in accordance with the deviance and assigns the best alternative when a preference is modest, and when a divergence is minuscule, there is no preference. The relationship between deviation and preference is linear. The actual figures between 0 and 1 represent the preferences. The best alternative from the available alternatives is chosen using a decision matrix. It serves as a measuring stick for how well each criterion was performed. Different decision support systems are effective at calculating and analyzing alternatives with the same or different criteria while employing the PROMETHEE-II technique.

### PROMETHEE-II

The following are the steps of the PROMETHEE-II technique^56,57^:

Phase 1: **Problem Space**- To determine the problem’s dimension, a decision matrix with alternatives as **I** (inhibitors) and criteria as **GF** (Growth Factors) is selected, along with the nature and weight of each criterion.

Phase 2: **Normalization**- The Decision Matrix can be normalized using the minimum-maximum methodology. Both direct and indirect criteria can be used to perform this normalization, where Eq. (1) is used for beneficial criteria and Eq. (2) for non-beneficial or cost criteria.

1 $$\begin{aligned} Min S_{ij}= & {} \frac{\left[ C_{ij}-min\left( C_{ij}\right) \right] }{\left[ max\left( C_{ij}\right) -min\left( C_{ij}\right) \right] } \end{aligned}$$

2 $$\begin{aligned} Max S_{ij}= & {} \frac{\left[ max\left( C_{ij}\right) -C_{ij}\right] }{\left[ max\left( C_{ij}\right) -min\left( C_{ij}\right) \right] } \end{aligned}$$

Here, i=1, 2, 3....m and j=1, 2, 3....n

Phase 3: **Pair-by-pair comparison**- For each criterion, determine the evaluation differences $w_j$ between the alternatives $i^{th}$ in relation to other alternatives. The alternative’s pairwise comparison is determined using Eq. (3).

3 $$\begin{aligned} w_j(z,y) = t_j(z)-t_j(y) \end{aligned}$$

Phase 4: **Calculation of the value of preference**- There are six preference functions in the PROMETHEE technique. Researchers have utilized V-shape with indifference criteria among six of the preference functions in this case. This procedure is always given 0 when the evaluated value of criteria is negative or equal to zero.

4 $$\begin{aligned} P_j(w) = \Bigg \{\begin{array}{c} t_j(z)-t_j(y); \textrm{w}>0 \\ 0; \quad \quad \quad \textrm{w}<=0 \quad \quad \end{array} \end{aligned}$$

Phase 5: **Aggregated preference value**- When the weights of $d_j$ are satisfied, Eq. (5) evaluates the preference aggregated value.

5 $$\begin{aligned} \left\{ \begin{array}{c} \pi (z,y)= \displaystyle \sum _{j=1}^{k}P_j(z,y)d_j,\\ \pi (y,z)= \displaystyle \sum _{j=1}^{k}P_j(y,z)d_j,\\ \sum _{j=1}^{k}d_j=1 \qquad \qquad \qquad \\ \end{array}\right. \end{aligned}$$

**Figure 3:**
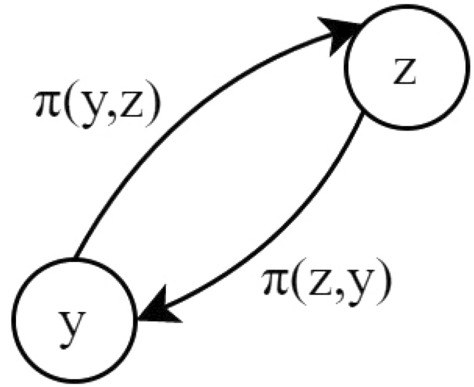
Valued Outranking Graph.

After computing $\pi (z,y)$ and $\pi (y,z)$ for each pair, a full-valued outranking graph is produced, with two arcs connecting each pair of nodes as shown in “Fig. 3”.

Phase 6: **The positive and negative outranking flow**- By calculating outranking flows, each alternative is connected to the other. The $z^{th}$ alternatives’ positive and negative outranking flows are estimated by Eqs. (6) and (7), respectively.

6 $$\begin{aligned} \phi ^+(z)= & {} \frac{1}{n-1}\displaystyle \sum _{x\epsilon z}\pi (z,y) \end{aligned}$$

7 $$\begin{aligned} \phi ^-(z)= & {} \frac{1}{n-1}\displaystyle \sum _{x\epsilon z}\pi (y,z) \end{aligned}$$

**Figure 4:**
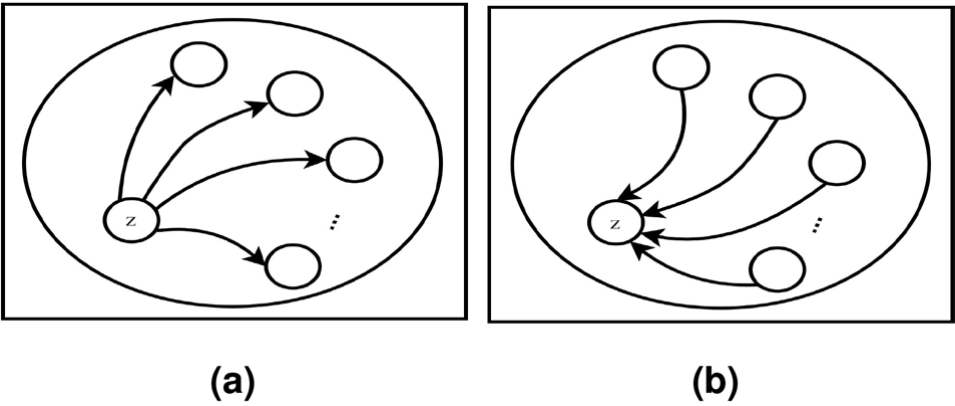
The $\phi ^+(z)$ and $\phi ^-(z)$ Outranking Flow.

The positive/ leaving flow describes an alternative’s outranking over all other alternatives, this is due to its dominating character and power as shown in “Fig. 4a”. Whereas the negative/ entering flow illustrates an alternative’s outranked over all other alternatives, this is due to its dominated character and weakness as shown in “Fig. 4b”. As, the higher value of $\phi ^+(z)$ and lower value of $\phi ^-(z)$, provides a better alternative.

Phase 7: Determine the **Net outranking flow** for each alternative using equation (8).

8 $$\begin{aligned} \phi (z)= \phi ^+(z)-\phi ^-(z) \end{aligned}$$

## Numerical analysis

Angiogenesis emanates from a disequilibrium of several pro- and anti-angiogenic endogenous factors that might contribute to ailment progression. The effective therapeutic approach is anti-angiogenesis inhibitors, which aim to inhibit the growth of neovascularization and deprive tumors of oxygen and nutrition. In this study, researchers have identified growth factors of angiogenesis in cancer, which is the chief factor. It serves as a defining factor of cancer as it is essential for both disease progression and metastasis. The following linguistic phrases and crisp scores were utilized on the 5-point fuzzy verbal scale: High (1.000), Average (0.666), Low (0.333), Very Low (0.250), and Null (0.000). This study has taken, the growth factor as criteria that ought to be maximized and inhibitors as alternatives for qualitatively rank assessment using a fuzzy verbal scale. In order to accumulate the data for this study, 72 experts from academia and the healthcare sector contributed their perspectives through Questionnaire in Linguistic form. The answer provided by the experts includes both qualitative and quantitative performance for each particular criterion. The authors have transformed the linguistic values into numerical values. Once the numerical values are estimated based on alternatives and criteria, the Decision matrix (DM) has been defined in Table 3. This table depicts how the DM judged the assessment of the alternatives.

**Table 3:** Decision Matrix.

Criteria / Alternative	GF1	GF2	GF3
$In_1$	0.333	0.250	0.333
$In_2$	0.333	0.250	0.333
$In_3$	1.000	0.000	0.333
$In_4$	1.000	0.666	0.666
$In_5$	0.333	0.000	0.333
$In_6$	1.000	0.333	0.666
$In_7$	0.666	0.000	0.333
$In_8$	1.000	0.250	0.333
$In_9$	1.000	1.000	0.333

The fuzzy scale was applied to convert information from the decision matrix into crisp values by using equation (1) to evaluate the normalized data as beneficial criteria shown in Table 4. Additionally, the DM further assigned relative weights that were normalized between 0 and 1 such as GF1 = 0.45, GF2=0.35, and GF3= 0.20. Using this criteria weight, Table 5 exhibits the matrix of preference function for the alternative pairs using equations (3) and (4) represented in 72 pairs of alternatives for estimating the differences among other alternatives.

**Table 4:** Normalized Decision Matrix.

Criteria / Alternative	GF1	GF2	GF3
$In_1$	0	0.250	0
$In_2$	0	0.250	0
$In_3$	1	0	0
$In_4$	1	0.666	1
$In_5$	0	0	0
$In_6$	1	0.333	1
$In_7$	0.499	0	0
$In_8$	1	0.250	0
$In_9$	1	1	0

**Table 5:** Preference Matrix of Alternative Pairs.

Criteria / Alternative	GF1	GF2	GF3
S($In_1$– $In_2$)	0	0	0
S($In_1$– $In_3$)	0	0.250	0
S($In_1$– $In_4$)	0	0	0
S($In_1$– $In_5$)	0	0.250	0
S($In_1$– $In_6$)	0	0	0
S($In_1$– $In_7$)	0	0.250	0
S($In_1$– $In_8$)	0	0	0
S($In_1$– $In_9$)	0	0	0
S($In_2$– $In_1$)	0	0	0
S($In_2$– $In_3$)	0	0.250	0
S($In_2$– $In_4$)	0	0	0
S($In_2$– $In_5$)	0	0.250	0
S($In_2$– $In_6$)	0	0	0
S($In_2$– $In_7$)	0	0.250	0
S($In_2$– $In_8$)	0	0	0
S($In_2$– $In_9$)	0	0	0
S($In_3$– $In_1$)	1	0	0
S($In_3$– $In_2$)	1	0	0
S($In_3$– $In_4$)	0	0	0
S($In_3$– $In_5$)	1	0	0
S($In_3$– $In_6$)	0	0	0
S($In_3$– $In_7$)	0.501	0	0
S($In_3$– $In_8$)	0	0	0
S($In_3$– $In_9$)	0	0	0
S($In_4$– $In_1$)	1	0.416	1
S($In_4$– $In_2$)	1	0.416	1
S($In_4$– $In_3$)	0	0.666	1
S($In_4$– $In_5$)	1	0.666	1
S($In_4$– $In_6$)	0	0.333	0
S($In_4$– $In_7$)	0.501	0.666	1
S($In_4$– $In_8$)	0	0.416	1

S($In_4$–$In_9$)	0	0	1
S($In_5$–$In_1$)	0	0	0
S($In_5$– $In_2$)	0	0	0
S($In_5$– $In_3$)	0	0	0
S($In_5$– $In_4$)	0	0	0
S($In_5$–$In_6$)	0	0	0
S($In_5$– $In_7$)	0	0	0
S($In_5$– $In_8$)	0	0	0
S($In_5$– $In_9$)	0	0	0
S($In_6$– $In_1$)	1	0.083	1
S($In_6$– $In_2$)	1	0.083	1
S($In_6$– $In_3$)	0	0.333	1
S($In_6$– $In_4$)	0	0	0
S($In_6$– $In_5$)	1	0.333	1
S($In_6$– $In_7$)	0.501	0.333	1
S($In_6$– $In_8$)	0	0.083	1
S($In_6$– $In_9$)	0	0	1
S($In_7$– $In_1$)	0.499	0	0
S($In_7$– $In_2$)	0.499	0	0
S($In_7$– $In_3$)	0	0	0
S($In_7$– $In_4$)	0	0	0
S($In_7$– $In_5$)	0.499	0	0
S($In_7$– $In_6$)	0	0	0
S($In_7$– $In_8$)	0	0	0
S($In_7$– $In_9$)	0	0	0
S($In_8$– $In_1$)	1	0	0
S($In_8$– $In_2$)	1	0	0
S($In_8$– $In_3$)	0	0.250	0
S($In_8$– $In_4$)	0	0	0
S($In_8$– $In_5$)	1	0.250	0
S($In_8$– $In_6$)	0	0	0
S($In_8$– $In_7$)	0.501	0.250	0
S($In_8$– $In_9$)	0	0	0
S($In_9$– $In_1$)	1	0.750	0
S($In_9$– $In_2$)	1	0.750	0
S($In_9$– $In_3$)	0	1	0
S($In_9$– $In_4$)	0	0.334	0
S($In_9$– $In_5$)	1	1	0
S($In_9$– $In_6$)	0	0.667	0
S($In_9$– $In_7$)	0.501	1	0
S($In_9$– $In_8$)	0	0.750	0

Thereafter, the assessment of preference function, the aggregate function is evaluated with the help of Eq. (5), quantified in Table 6, representing the preferable ranking between two inhibitors based on the three criteria. Later Eqs. (6–8) evaluates the positive and negative ranking flow to analyze the net outranking flow to estimate the inhibitors ranking among in shown in Table 7.

**Table 6:** Aggregated Preference Value.

	$In_1$	$In_2$	$In_3$	$In_4$	$In_5$	$In_6$	$In_7$	$In_8$	$In_9$
$In_1$	$\cdots$	0	0.088	0	0.088	0	0.088	0	0
$In_2$	0	$\cdots$	0.088	0	0.088	0	0.088	0	0
$In_3$	0.450	0.450	$\cdots$	0	0.450	0	0.225	0	0
$In_4$	0.796	0.796	0.433	$\cdots$	0.883	0.117	0.659	0.346	0.200
$In_5$	0	0	0	0	$\cdots$	0	0	0	0.000
$In_6$	0.679	0.679	0.317	0	0.767	$\cdots$	0.542	0.229	0.200
$In_7$	0.225	0.225	0	0	0.225	0	$\cdots$	0	0
$In_8$	0.450	0.450	0.088	0	0.538	0	0.313	$\cdots$	0
$In_9$	0.713	0.713	0.350	0.117	0.800	0.233	0.575	0.263	$\cdots$

**Table 7:** Positive, Negative Ranking Flow, and Net Outranking Flow.

	$\phi ^+$	$\phi ^-$	$\phi$(Total)	Rank
$In_1$	0.033000	0.414125	$-0.38113$	7
$In_2$	0.033000	0.414125	−0.38113	8
$In_3$	0.196875	0.170500	0.026375	5
$In_4$	0.528750	0.014625	0.514125	1
$In_5$	0	0.383900	−0.383900	9
$In_6$	0.426625	0.043750	0.4391250	2
$In_7$	0.084375	0.311250	−0.226880	6
$In_8$	0.229875	0.104750	0.125125	4
$In_9$	0.470500	0.050000	0.420500	3

**Figure 5:**
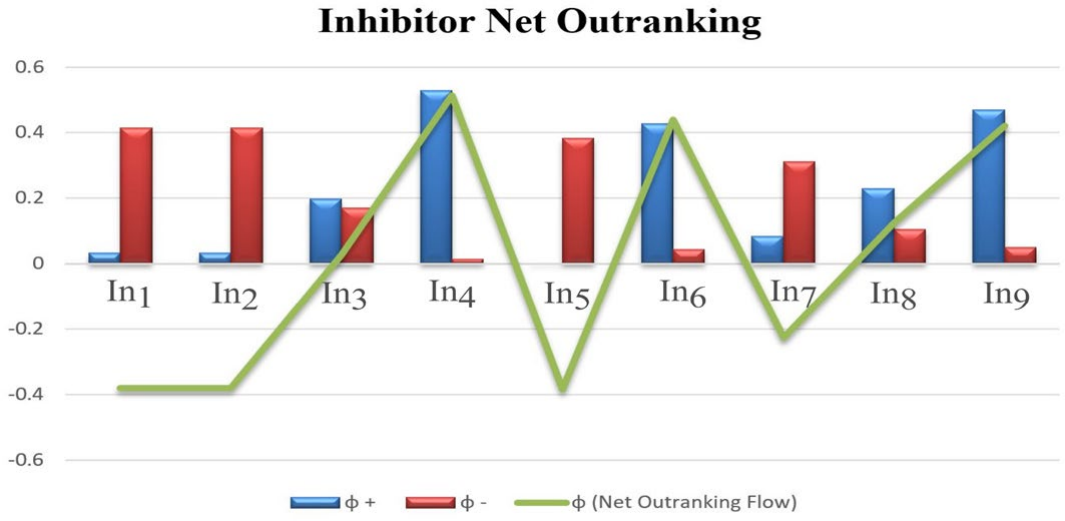
Inhibitor Net Outranking Flow with Positive and Negative Ranking.

In the final column, the ranking of the best and least alternative is shown in “Fig. 5”. Evaluation findings indicate that alternative I4 ranked as the most efficacious alternative for inhibiting angiogenesis in cancer, having the highest $\phi (i)$ of the three factors **GF1**, **GF2**, and **GF3** that were examined. Nine anti-angiogenic inhibitors of cancer $In_4$, $In_6$, $In_9$, $In_8$, $In_3$, $In_7$, $In_1$, $In_2$, and $In_5$ were administered consecutively to assist medical organizations or decision-makers to adopt or optimize respective medical interventions.

## Conclusion

Cancer research is one of the fast-emerging sectors in the development of novel compounds that target molecules for the treatment of specific diseases. A significant breakthrough in cancer research has been made possible by improving cancer immunity and identifying the receptor that reduces or blocks cancer cells. The most promising cancer treatment is immunotherapy, which blocks the aberrant proteins that rapidly trigger cells to develop tumors. An imbalance among various anti-angiogenic and pro-angiogenic endogenous factors leads to angiogenesis, which may cause the evolution of the disease. Angiogenesis acts as an important mechanism for immune evasion. The multi-stage process of angiogenesis, which comprises the migration, proliferation, and capillary development of EC, is a critical factor for the development and metastasis of tumors. The pro-angiogenic factors are responsible for the growth of tumor cells, it causes tumor growth by the diffusion of nutrients and oxygen from proximal capillaries. In this study, the authors discussed three common growth factors that involve abnormal cell growth and have the potential to spread or invade different body parts. VEGF, FGF, and PDGF are common growth factors discussed in this paper, and any deviations in their normal function led to various abnormalities. These factors normally function as extracellular molecules that circulate locally or systemically and activate cell surface receptors involved in angiogenesis and anti-angiogenic therapies. The RTKs have high-affinity cell surface receptors, kinase inhibitor resistance represents an important unmet clinical need in cancer. This research investigated nine anti-tumor or anti-angiogenic inhibitors based on VEGF, FGF, and PDGF receptors. Using the PROMETHEE-II method, the authors evaluate the rank assessment of anti-angiogenic inhibitors. This technique is based on a multi-criteria analysis that estimates the impacts of several factors to predict good decision results. As per the PROMETHEE- II investigation, the BIBF 1120 inhibitor is most efficacious as an anti-angiogenic inhibitor followed by CHIR-258, E7080, GW786034 HCl, BAY73-4506, Bay43-9006, AP24534, SU 5402, and ABT-869. This study shows that the suggested techniques present sophisticated alternate solutions to decision-making challenges straightforwardly and practically. The results of this study will help medical institutes and researchers in making pertinent decisions for selecting an inhibitor, moreover, the approach can be applied to various aspects of medical decision-making. In the future, the authors of this study will examine a wide range of anti-angiogenesis inhibitors that may result in the most favorable outcomes.

## Data Availability

The datasets used and analyzed during the current study available from the corresponding author on reasonable request.
